# Digital Omicron detection using unscripted voice samples from social media

**DOI:** 10.1101/2022.09.13.22279673

**Published:** 2022-12-22

**Authors:** James T. Anibal, Adam J. Landa, Hang T. Nguyen, Alec K. Peltekian, Andrew D. Shin, Miranda J. Song, Anna S. Christou, Lindsey A. Hazen, Jocelyne Rivera, Robert A. Morhard, Ulas Bagci, Ming Li, David A. Clifton, Bradford J. Wood

**Affiliations:** 1. Center for Interventional Oncology, Radiology and Imaging Sciences, NIH Clinical Center, National Cancer Institute, National Institute of Biomedical Imaging and Bioengineering, National Institutes of Health, 10 Center Dr, Building 10, Room 1C341, MSC 1182, Bethesda, MD 20892-1182 USA; 2. Computational Health Informatics Lab, Oxford Institute of Biomedical Engineering, University of Oxford, Old Road Campus Research Building, Headington, Oxford OX3 7DQ, United Kingdom; 3. Oxford University Clinical Research Unit, Centre for Tropical Medicine, 764 Vo Van Kiet, Quan 5, Ho Chi Minh City, Vietnam; 4. Department of Computer Science, McCormick School of Engineering, Northwestern University, Mudd Hall, 2233 Tech Drive, Third Floor, Evanston, IL, 60208 USA; 5. National Library of Medicine, National Institutes of Health, 8600 Rockville Pike, Bethesda, MD, 20894; 6. Feinberg School of Medicine, Northwestern University, 420 E Superior St, Chicago, IL 60611 USA

## Abstract

The success of artificial intelligence in clinical environments relies upon the diversity and availability of training data. In some cases, social media data may be used to counterbalance the limited amount of accessible, well-curated clinical data, but this possibility remains largely unexplored. In this study, we mined YouTube to collect voice data from individuals with self-declared positive COVID-19 tests during time periods in which Omicron was the predominant variant^1,2,3^, while also sampling non-Omicron COVID-19 variants, other upper respiratory infections (URI), and healthy subjects. The resulting dataset was used to train a DenseNet model to detect the Omicron variant from voice changes. Our model achieved 0.85/0.80 specificity/sensitivity in separating Omicron samples from healthy samples and 0.76/0.70 specificity/sensitivity in separating Omicron samples from symptomatic non-COVID samples. In comparison with past studies, which used scripted voice samples, we showed that leveraging the intra-sample variance inherent to unscripted speech enhanced generalization. Our work introduced novel design paradigms for audio-based diagnostic tools and established the potential of social media data to train digital diagnostic models suitable for real-world deployment.

## 1. Introduction

COVID-19 is routinely detected and confirmed through polymerase chain reaction (PCR) using nasal or throat swabs; however, turnaround time and resource costs pose a challenge for testing in some settings. Some invasive home testing methods have been developed but can require expensive reagents and/or laboratory expertise, restricting accessibility. Moreover, these tests do not offer immediate results, which have become increasingly necessary as societies move towards “living with COVID”^4^. Serial testing practices have become common, in which reasonably sensitive at-home antigen tests are followed by more-specific PCR confirmation. Instant, non-invasive, and sensitive testing methods may be useful for suggesting confirmatory testing or to track the spread of variants with unique audio phenotypes.

Prior AI methods have been unable to successfully detect pre-Omicron variants from unscripted or scripted human voice alone, or have been otherwise unsuitable for deployment (e.g., limited training data, poor generalization)^5^. Omicron variants, however, are typically milder and affect the upper airway more commonly than prior variants, often resulting in voice changes without a cough or other respiratory symptoms^6^. This presents an opportunity for targeting with AI methods, if robust datasets were available.

Worldwide, various social media platforms have over 3.6 billion users, with expectations to exceed 4.4 billion by 2025^7^. Over 500 hours of video are uploaded to YouTube alone every minute^8^. While often ignored, much of this data is available to researchers through Python libraries provided by social media companies. Such data more accurately portrays noisy, unscripted “real-world” data, whose broad diversity supports generalizability. Our model was trained using this freely accessible data. Though annotation of training data was based upon prevalence assumptions and self-declaration instead of sequencing, these limitations contributed to the practicality and cost-effectiveness of the approach. As opposed to other methods that rely on lab results, AI-based classification tools using voice alone could be instant, accessible, cost-effective, and deployable in real-world settings.

There are numerous barriers to practical deployment of AI models for COVID-19 diagnostics. Prior attempts have failed due to training on extremely limited datasets, producing overfit models that do not generalize^9^. Existing models for acoustic, AI-driven diagnostics have also been limited due to a reliance on short, structured samples collected in controlled, scripted environments. In this report, we broadly applied dataset design and audio-based deep learning methodologies in the context of Omicron classification.

### Contributions:

1. An AI system was constructed using social media data, with training and validation strategies designed for real-world clinical settings. This improves upon prior social media AI efforts which simply facilitated narrow tasks in controlled settings involving frequent users of social media.
2. A new framework for rapid diagnostic tools from voice/audio data was designed to emphasize longer samples and unscripted collection protocols. This strategy relied on the diversity of the input data to better prepare for real-world testing environments. A convolutional neural network (CNN) model trained on long, unscripted samples showed improved generalization in comparison to prior work on short, scripted speaking inputs, as well as in our comparative experiments involving short, unscripted inputs.
3. The first non-invasive model for instant Omicron detection was developed using training data that included past variants, recent subvariants, healthy controls, and other respiratory illnesses. Our data was compiled from a diverse array of settings and recordings, containing over 28 hours of unscripted audio from people posting with self-declared Omicron. This is several orders of magnitude greater than previous efforts. Identification of voice changes in speakers with self-declared Omicron showed that unscripted recordings may be sufficient for detecting Omicron COVID-19, which is a change from past variants.
4. Acoustic biomarkers for COVID-19 were shown to change between non-Omicron (primarily Alpha/Delta) variants (lower respiratory symptoms) and the milder Omicron variant (upper respiratory symptoms). Models trained on voice data from pre-Omicron variants experienced a decrease in both sensitivity and specificity when validated on Omicron test data. This result emphasized the necessity of **variant-specific methods for testing**, or continual learning techniques that adapt as pandemics evolve over time.

## 2. Related Work

### 2.1 Social Media Data for Clinical Tasks

Cost-effective data collection, curation, annotation, and augmentation are critical for enabling AI to track or predict illness. Social media is an expansive source of information that does not rely on intricate searching mechanisms or filtered, delayed reporting, potentially facilitating epidemiological surveillance.

Several existing diagnostic models have utilized social media data. A deep learning model trained on Tweets was more predictive of atherosclerotic heart disease mortality than a conventional mechanistic input-based model combining demographic and health risk factors^10^. Additional methods have extracted textual and visual features from Tweets to predict and classify mental health status^11,12,13^. These models, however, were only useful for active social media users. Other social media platforms have also been used as data sources for biomedical applications, though less frequently than Twitter. Manual analysis of YouTube home videos by non-clinical raters was able to detect and classify autism in children with high performance^14^. YouTube audio, visual, and search-history data have also successfully detected mental illnesses including depression and OCD^15,16^.

### 2.2 AI for COVID-19 Testing

AI methodologies have been applied to numerous COVID-19 datasets to develop deployable diagnostic tools. Coughing and breathing changes associated with COVID-19 may have unique features that might be useful for classification, or differentiation from other upper respiratory illnesses or infections (URIs). Related work should, however, be contextualized using the criteria outlined by Han et al., which pointed out methodological flaws such as mixing training/testing data, exclusion of non-COVID URIs, and overfitting on small datasets^5^. Prior work has also typically lacked stratification by variant, instead considering “COVID” as a whole and assuming a generally consistent phenotype.

Nonetheless, previous efforts highlighted the potential for voice-based diagnostic tools, especially given that COVID-19 breath sounds were characterized by unique time and frequency domain patterns^17^. A CNN-based model trained on forced-cough recordings in limited numbers of patients with and without COVID-19 was able to recognize COVID-19 with high sensitivity, even in otherwise asymptomatic subjects^18^. Audio-based technologies using cough sounds have also been deployed on a smartphone app for COVID-19 detection^19,20,21,22,23^. A binary classifier was able to differentiate COVID-19 speech from normal speech based on scripted telephone data^24^. Assessment of spectral features of speech alone in asymptomatic patients with and without COVID-19 yielded a true positive rate of 70%, though the likelihood of generalization was quite limited due to scripted collection and small sample size.^25^

In a dynamic pandemic such as COVID-19, crowdsourced datasets allowed for continuous and focused sample collection. “Coswara” is a database containing COVID-19 respiratory sound samples, including cough, breath, and scripted voice data^26^. Samples recorded and uploaded by volunteers on a smartphone or computer were divided into COVID and non-COVID cohorts^27^. Numerous researchers have used this database to train AI models for COVID-19 detection^20,23,28^. Such algorithms reported an accuracy of 97% on limited binary datasets, which notably excluded other respiratory illnesses^29,30^. Deep learning models trained on the “Sounds of COVID” dataset, which contained scripted samples, showed that voice alone performed poorly on pre-Omicron data (0.61 ROC-AUC)^5^. Further, most studies focused on multi-input models without specifying the variant (via sequencing or demographic statistics).

## 3. Methods

**Figure 1:**
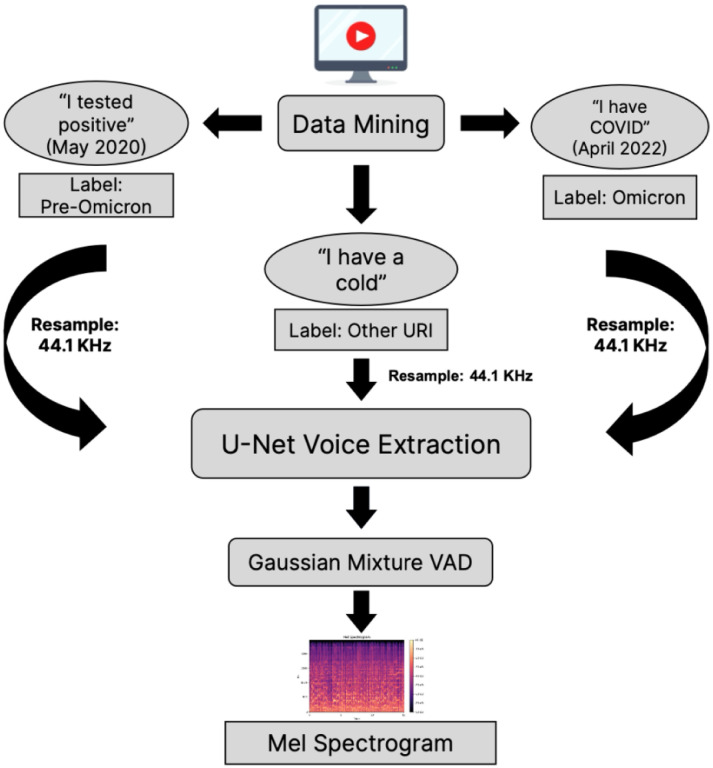
Workflow for YouTube data preprocessing. (1) Data was collected from YouTube and annotated based on user declarations. (2) A U-Net model was used to separate voice from music and other background noise. (3) A voice activity detector (VAD), built with Gaussian mixture models removed extended periods of silence. (4) The remaining, cleaned voice data was converted into a spectrogram.

**Figure 2:**
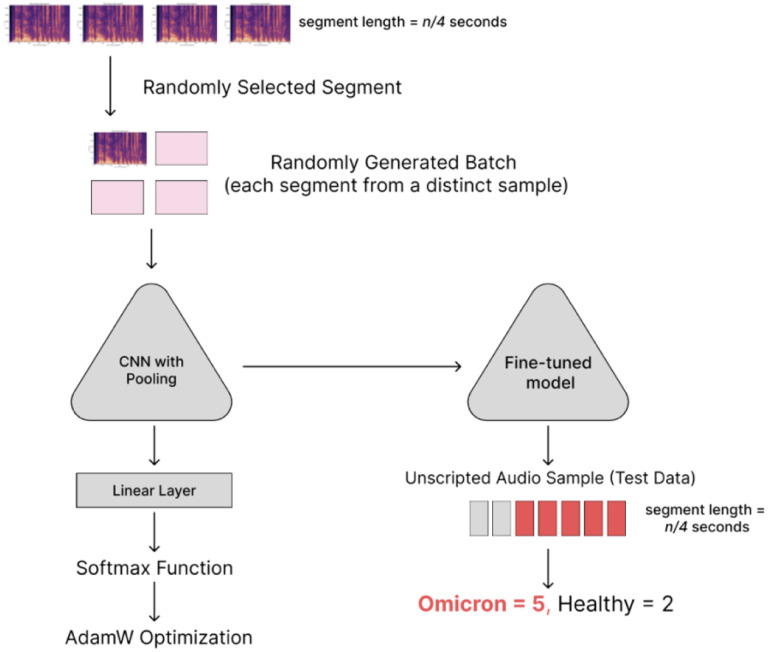
Workflow for training and validation of Omicron detection pipeline. (1) Audio recordings were split into segments and converted to spectrograms; (2) DenseNet model was trained on the spectrograms; (3) trained model was used to predict if segments in a testing dataset were positive or negative for Omicron COVID-19 (majority vote was used to assign a label).

The methods were performed in accordance with relevant guidelines and regulations and approved by the Institutional Review Board (IRB) at the NIH. The preprocessing, training, and validation components of the Omicron detection method are outlined in Fig. 1–2.

### 3.1 Data Collection

Audio samples were mined from YouTube searches and annotated by cohort based on self-declaration and presumptive correlation with epidemiological data.

1. COVID-19 – Omicron variant (presumed by dates, including both symptomatic and asymptomatic cases)
2. COVID-19 – non-Omicron variant (presumed by dates, including both symptomatic and asymptomatic cases)
3. Symptomatic, non-COVID upper respiratory illnesses and infections (URI)
4. Presumably healthy or non-acutely ill (asymptomatic).

A series of heuristics were used to identify relevant videos. For example, if the user said, “I have COVID” or “I tested positive” during a time in which Omicron was the dominant variant, the audio sample was labeled as “Omicron”. We excluded videos that contained low-quality audio or featured multiple speakers. For the URI cohort, we excluded videos where there were no obvious respiratory symptoms. YouTube videos were annotated by cohort and manually verified to ensure accurate labeling. Since the data mining procedure was standardized but not comprehensive, future studies might benefit from automated mining procedures for dataset expansion. All Omicron videos were from December 20^th^, 2021 – August 1^st^, 2022. Omicron was designated a “variant of concern” on November 26^th^, 2021 by the WHO, and was estimated to be the dominant variant in the US by late December 2021^31,32^. Omicron was identified as the dominant variant globally, accounting for > 98% of sequences shared on GISAID after February 2022^2,33^. The BA.1 and BA.2 lineages were most common between December 2021 – June 2022, with BA.4/BA.5 becoming more prevalent in July 2022^34^. No sequencing was recorded.

### 3.2 Data Preprocessing

Raw audio samples extracted from YouTube videos were noisy, often containing background noise, long periods of silence, or low-resolution audio. To reduce potential sources of confusion, a preprocessing pipeline was implemented:

1. **Audio Denoising:** Following audio quality assessments, “noisy” sound was removed using semi-supervised machine learning methods developed by Dolby and accessed through Dolby Media libraries for Python^35^.
2. **Removal of Background Noise and Silence:** Background noise was removed via a U-Net convolutional neural network architecture^36^. Extended periods of silence were removed via a voice activity detector that leveraged Gaussian mixture models to identify non-speaking regions^37^.
3. **Conversion into Mel spectrograms:** Samples were converted into a 3-channel matrix, corresponding to 3 Mel spectrograms generated with different window sizes and hop lengths. Mel spectrograms represented sound as frequency over time with frequency values converted to the Mel scale, which represents pitch based on how the human ear perceives loudness. This approach ensured that each channel contained different frequency and time information (equivalent to resolution in a standard image representation), providing the model with maximal context during training^38^.

### 3.3 Augmentation

In audio-based diagnostics, there is minimal value in positional context. While past work has shown that sounds have variable effectiveness as disease predictors, we further assumed that relevant digital biomarkers of laryngitis should be detectable within a 10 second interval^39^. Our simple data augmentation strategy relied on the positional invariance of speech samples and the reduced need for modeling long-term dependencies, in the context of laryngitis. We aimed to use the natural diversity of speech to enhance the generalizability of our model and reduce the impact of class imbalance. For each audio recording, we considered the set of possible transformations to be the result of dividing the sample into segments of length *n* seconds. Time and frequency masking were applied to each segment (via SpecAugment) prior to input into the CNN model, which is explained in section 3.4^40^.

### 3.4 DenseNet

Convolutional neural networks (CNNs) utilize the convolution operation to model spatial relationships in matrices (e.g., images or spectrograms). These representations are generally input into a standard feed-forward neural network and mapped onto an outcome or embedding vector. In most cases, individual layers are connected only to the subsequent layer. DenseNet introduced a new framework wherein each layer was connected to all subsequent layers in a “Dense Block”, while allowing for multiple Dense Blocks within the same network^41^. These blocks were connected to each other via convolution and pooling layers which structured the outputs of one block as inputs for the following block. This approach had multiple advantages for complex tasks, including improved feature propagation and reuse.

The DenseNet model was chosen due to the scalability of the architecture and high top-1 accuracy value on the complex ImageNet dataset, indicating that it had learned a generalizable representation of images through key shapes/features. A pre-trained model was chosen based on prior work which reported that CNN models pre-trained on ImageNet achieved superior performance on audio data compared to randomly initialized models^38,42^. Other recent architectures (e.g., Vision Transformer) can also be used in our model-invariant system^43^.

## 4. Experimental Design

Experiments were performed to assess the potential of social media data for training models to complete diagnostic tasks. We also assessed the generalization capacity gained from using the long, free-response inputs as a component of the data augmentation strategy alongside SpecAugment (compared to short, standardized inputs). The reported performance metrics were mean values from 6-fold cross validation used in each experiment (Table 2).

### 4.1 YouTube Dataset

Our YouTube dataset contained 183 subjects with Omicron (28.39 hours), 120 with pre-Omicron COVID-19 (22.84 hours), 138 with symptomatic non-COVID URIs (8.09 hours), and 192 that were asymptomatic healthy (33.90 hours). Dataset statistics are listed in Table 1 to emphasize the additional information gained from collecting longer samples of unscripted voice data.

To the best of our knowledge, the YouTube dataset currently contains the largest amount of voice data (in hours) for COVID-19 (all variants), the Omicron variants specifically, and, particularly, URI that were confirmed or self-declared non-COVID. This is in comparison to all publicly available COVID-19 voice/sounds datasets. The Coswara dataset contained samples which may be other upper respiratory infections but were not confirmed or self-declared as non-COVID^25^. We also noted that nearly half the 1,964 negative participants in the “Sounds of COVID” dataset had at least one “COVID” symptom, but many were unrelated to the upper respiratory system (e.g., fever, dizziness)^5^.

In contrast, the YouTube dataset intentionally included illnesses that were designated non-Omicron based on self-declaration or date of posting. The samples were selected for the potential to impact the upper respiratory system, teaching the model to separate between non-COVID illnesses and Omicron. These URI included influenza, strep throat, cold, allergy attack, asthma, bronchitis, and others. Prior to training, we applied preprocessing and augmentation strategies to the dataset as described in sections 3.2–3.3.

### 4.2 DenseNet Model Training

For supervised classification tasks involving voice samples, a cross-entropy loss function was used to fine-tune a pre-trained DenseNet. To address imbalances in the dataset, each batch was generated by oversampling the minority class. For each sample in the batch, a 2.5 second voice segment was selected randomly from the entire audio recording.

#### 4.2.1 Classification of Omicron and Asymptomatic Healthy Subjects

A DenseNet model was trained to identify healthy subjects (e.g., for testing asymptomatic individuals prior to attending populated events) through the binary classification task of separating asymptomatic healthy voices from all Omicron subjects.

#### 4.2.1 Classification of Omicron and Symptomatic URI Subjects

For further classification of users presenting with upper-respiratory symptoms, the model was trained to use voice data to separate Omicron subjects from symptomatic subjects with other, non-COVID URIs.

#### 4.2.3 Model Training with Non-Omicron COVID-19 Data

We trained a model with non-Omicron data and attempted to classify COVID/URI and COVID/healthy from test sets containing only Omicron and non-COVID subjects (no other variants were present). This was done to highlight changes in disease phenotype over time and show the need for variant-specific digital testing methods.

#### 4.2.4 Model Training with Single Segments

To demonstrate the importance of collecting longer time-series datasets, we repeated the Omicron detection tasks (4.2.1–4.2.2) with short, randomly selected segments (one per sample) from the YouTube dataset. The same segment was used throughout the training process – the remainder of the data was not shown to the model. Due to model overfitting, we froze the base layers of the DenseNet and fine-tuned only the classification head.

### 4.3 Model Validation

When validating on a blind test dataset, sensitivity and specificity were calculated on a per-sample basis. Each sample was divided into *n*-second segments as described in section 3.3. For samples that could be split into multiple segments, a majority vote was used to assign the final label (“positive” or “negative”). This approach was used to facilitate real-world deployment where the user might be prompted to supply at minimum 30 seconds of audio, thereby reducing the risk of random noise or vocal shifts that could obscure relevant digital biomarkers.

## 5. Experimental Results

### 5.1 Digital Testing on the YouTube Dataset

#### 5.1.1 Extended Voice Samples

Our model was tested on the YouTube dataset to perform classification tasks (Table 2). Voice changes were used to detect the Omicron variant on “real-world” data from YouTube. Model performance was 85% specific and 80% sensitive for classification of Omicron subjects and asymptomatic healthy subjects. Notably, the model yielded a specificity of 76% and a sensitivity of 70% on the symptomatic testing task of separating Omicron samples from other, symptomatic URI samples. These findings indicate the existence of an Omicron-specific laryngitis. This finding suggests that models trained on real-world voice data may have relevance for symptomatic testing, beyond identifying healthy, asymptomatic voices.

Our model was further tested on the YouTube dataset to determine if voice changes caused by Omicron represented a detectable shift in phenotype compared to Alpha/Delta variants, which often presented with lower respiratory symptoms. Like past work, the results show that voice data alone does not facilitate the separation of pre-Omicron COVID-19 samples from asymptomatic healthy samples (84% specificity/ 58% sensitivity). The limited signal verifies the limited acoustic biomarkers in subjects with the pre-Omicron variants of COVID-19. The improved results on the COVID/URI classification task (74% specificity / 70% sensitivity) are likely due to the model detecting voice changes or other respiratory symptoms in the patients with URI. This is in contrast with the pre-Omicron COVID-19 patients, which seem to have similar voices to healthy asymptomatic subjects. Voice data was more effective at detecting the Omicron variant in both the healthy/COVID-19 classification task (a difference in sensitivity of over 20%), and the COVID-19/other URI classification task.

Furthermore, our results show that there is performance degradation for the Omicron/URI task (5–7% reduction in both sensitivity and specificity) when a non-Omicron model is validated on Omicron test data. This is in comparison to an Omicron-trained model that is validated on Omicron test data. The likely cause of this result is that the model does not learn to recognize the Omicron-specific laryngitis that may be an important feature for classification. There was a similar performance decline (5–10%) on the healthy/Omicron binary classification task (pre-Omicron training, Omicron testing), which is, once again, likely resulting from the model being boosted by the presence of “Omicron laryngitis” in the training data. We observe that the model for healthy/pre-Omicron classification achieves better performance on Omicron test data than the non-Omicron test data. We speculate that this is due to the limited presence of general laryngitis in the training data, which can be transferred to Omicron test data, where upper respiratory symptoms are common. For the non-Omicron test data, laryngitis in the test data, statistically, should be much more sporadic.

#### 5.1.2 Single-segment Voice Samples

We trained our model to complete both the Omicron detection tasks on the same subjects, with only one segment per subject in the dataset. Despite the use of multiple data augmentation techniques, our results (Table 3) showed a decrease in both sensitivity and specificity (10–20%) when only a single segment was used, demonstrating the importance of collecting longer time-series data, even if there are a limited number of subjects. In addition, these extended data points may reduce the risk of error in a real-world digital testing solution. When performing inference, the model can check multiple independent segments over a 30+ second input (faster than current rapid testing solutions) to reduce the risk of testing on a corrupted or unrepresentative data point.

## 6. Discussion

In this report, we show that:

1. Public online data, including unscripted social media data, had potential epidemiologic value in pandemics, with audio information that could be utilized by AI models for applications not specific to social media/Internet users.
2. In contrast with past variants, voice change was a predictor of the Omicron variant, which was often milder and lacked a cough. Omicron samples were distinguishable not only from healthy voices, but also from voices with other self-declared upper respiratory illness and infections.
3. Models trained on pre-Omicron data alone showed a decline in performance when validated on test datasets containing Omicron samples. This demonstrates the importance of testing methods which are continuously updated and validated with variant-specific data. This is relevant for future pandemic preparedness, where perhaps deep learning may play a more meaningful role.
4. Models trained on longer, unscripted audio samples achieved superior performance compared to shorter scripted inputs used in prior work (e.g., counting to 20) and shorter unscripted inputs (section 5.1.2). Lengthy voice samples improved model performance due to the diversity of unscripted data.

We introduced the “YouTube COVID-19 voice dataset”, which contains over one full day of audio data corresponding to the Omicron variant, and similar quantities for non-Omicron COVID-19 and healthy controls. The dataset also includes over 8 hours of data from other upper respiratory illness and infection, improving upon other COVID-19 audio datasets which were noisy, unbalanced, and contained undiagnosed URI data (unconfirmed COVID negativity). This comparison underscores the value of retrospective data collection from public social media in real-world settings, despite the lack of ground truth verification. Voice changes may represent a biomarker for Omicron. The exact underlying mechanism for characteristic audiogram alterations may be due to local laryngitis; however, the central nervous system is also capable of controlling pitch in speech^44,45^.

### 6.1 Real-World Deployment

Potential future directions for this work include dataset expansion through improved mining methods, implementation of a smartphone/web-app system for day-to-day testing or user-specific customized models, and development of continual learning. Furthermore, regular pre-screening in at-risk populations could be used to define the temporal/geographic dynamics of voice changes from variants as a virus evolves. However, model deployment, stability, and impact currently remain highly speculative due to the limited size of samples from other URIs, lack of PCR confirmation testing and sequencing, and cohort annotations based on assumption.

## 7. Conclusion

Digital epidemiology is provocative and understudied in the context of public online data and social media data; however, its wide availability raises new questions for privacy security, regulation, ethics, and real-world validation. Unscripted social media audio data may inherently be more diverse (and ultimately lead to more generalizable models) than narrow-intent scripted data. For symptomatic illnesses, these indicators may be more accurately reflected in longer samples. Even without ground truth from sequencing, the results achieved by this early effort at Omicron detection merit further evaluation or smartphone app assessment in specific controlled public health settings with unmet needs. Despite limitations, this work highlights the unique presentation of laryngitis in patients with the COVID-19 Omicron variant. Social media as a source of unscripted audio data may enable new frameworks to facilitate variant-specific testing.

## Figures and Tables

**Table 1:** Comparative statistics for two common COVID-19 Voice Datasets. Statistics from Coswara were listed based on the amount of data at the time of access (May 18^th^, 2022). We also excluded any data points which did not meet the criteria for inclusion in the Coswara database (e.g., multiple voices, indetectable audio).

Dataset	COVID-19 Samples (All)	Omicron Samples	Other URI (Symptomatic) Samples	COVID-19 total audio (hours)	Omicron total audio (hours)	URI total audio (hours)
YouTube Dataset	316	183	138	**51.23**	**28.39**	**8.09**
Coswara	464	**213**	102	1.92	0.95	0.47

**Table 2:** Model performance on COVID-19 detection (including both Omicron data and non-Omicron data) with randomly selected test datasets from the YouTube dataset.

Task	Train Data	Test Data	Specificity	Sensitivity
Asymptomatic Healthy vs. Omicron	Omicron	Omicron	0.85	0.80
Asymptomatic Healthy vs. Omicron	Non-Omicron COVID-19	Omicron	0.82	0.71
Omicron vs. Symptomatic URI	Omicron	Omicron	0.76	0.70
Omicron vs. Symptomatic URI	Non-Omicron COVID-19	Omicron	0.70	0.65
Asymptomatic Healthy vs. pre-Omicron	Non-Omicron COVID-19	Non-Omicron COVID-19	0.80	0.58
pre-Omicron vs. Symptomatic URI	Non-Omicron COVID-19	Non-Omicron COVID-19	0.74	0.70

**Table 3:** Model performance on Omicron detection with a single segment per sample.

Task	Train Data	Test Data	Specificity	Sensitivity
Healthy vs. Omicron	Omicron	Omicron	0.73	0.67
Omicron vs. URI	Omicron	Omicron	0.59	0.64

## Data Availability

Data will be made available for academic research upon requests directed to the corresponding email (james.anibal@reuben.ox.ac.uk).
